# Community Engaged Cumulative Risk Assessment of Exposure to Inorganic Well Water Contaminants, Crow Reservation, Montana

**DOI:** 10.3390/ijerph15010076

**Published:** 2018-01-05

**Authors:** Margaret J. Eggers, John T. Doyle, Myra J. Lefthand, Sara L. Young, Anita L. Moore-Nall, Larry Kindness, Roberta Other Medicine, Timothy E. Ford, Eric Dietrich, Albert E. Parker, Joseph H. Hoover, Anne K. Camper

**Affiliations:** 1 Center for Biofilm Engineering, Montana State University, P.O. Box 173980, Bozeman, MT 59717, USA; eric.dietrich@gmail.com (E.D.); parker@math.montana.edu (A.E.P.); acamper@montana.edu (A.K.C.); 2 Crow Environmental Health Steering Committee, Little Big Horn College, Crow Agency, MT 59022, USA; doylej@lbhc.edu (J.T.D.); myrajlefthand@gmail.com (M.J.L.); saralyoung@hotmail.com (S.L.Y.); kindnesslarry@yahoo.com (L.K.); roberta.othermedicine@ihs.gov (R.O.M.); 3 Crow Water Quality Project, P.O. Box 370, Little Big Horn College, Crow Agency, MT 59022, USA; 4 Department of Earth Sciences, Montana State University, P.O. Box 173480, Bozeman, MT 59717, USA; amoorenall@yahoo.com; 5 Environmental Health Department, Crow/Northern Cheyenne Indian Health Service Hospital, Crow Agency, MT 59022, USA; 6 School of Public Health and Health Sciences, University of Massachusetts Amherst, 715 N. Pleasant Street, Amherst, MA 01003, USA; teford@umass.edu; 7 Department of Mathematical Sciences, Montana State University, P.O. Box 173980, Bozeman, MT 59717, USA; 8 Health Sciences Center, MSC09 5360, 1 University of New Mexico, Albuquerque, NM 87131, USA; JoHoover@salud.unm.edu; 9 College of Engineering, Montana State University, P.O. Box 173980, Bozeman, MT 59717, USA

**Keywords:** drinking water, chemicals, health risks, exposure assessment, cumulative risk assessment, risk communication, CBPR, environmental health, environmental justice, Native American

## Abstract

An estimated 11 million people in the US have home wells with unsafe levels of hazardous metals and nitrate. The national scope of the health risk from consuming this water has not been assessed as home wells are largely unregulated and data on well water treatment and consumption are lacking. Here, we assessed health risks from consumption of contaminated well water on the Crow Reservation by conducting a community-engaged, cumulative risk assessment. Well water testing, surveys and interviews were used to collect data on contaminant concentrations, water treatment methods, well water consumption, and well and septic system protection and maintenance practices. Additive Hazard Index calculations show that the water in more than 39% of wells is unsafe due to uranium, manganese, nitrate, zinc and/or arsenic. Most families’ financial resources are limited, and 95% of participants do not employ water treatment technologies. Despite widespread high total dissolved solids, poor taste and odor, 80% of families consume their well water. Lack of environmental health literacy about well water safety, pre-existing health conditions and limited environmental enforcement also contribute to vulnerability. Ensuring access to safe drinking water and providing accompanying education are urgent public health priorities for Crow and other rural US families with low environmental health literacy and limited financial resources.

## 1. Introduction

Exposure to contaminants through unsafe drinking water is increasingly being recognized as contributing to environmental health disparities for rural, minority and other environmental justice communities in the United States [1,2,3,4,5,6]. These disparities take the form of denial of access to or poor quality municipal water, contaminated home well water or lack of in-home water service. Most existing research on these health disparities addresses community water and wastewater infrastructure [7,8,9,10,11,12,13,14,15,16,17,18,19,20,21]. However, disparities in access to safe drinking water from home wells for rural families in the United States (U.S.) remain poorly understood [22,23]. Although 15% of the US population (49 million people) rely on private wells, the federal government does not regulate home well water quality [24,25]. While some states require testing when new wells are drilled or when homes are sold [23], state laws do not apply on trust lands within most Indian reservations. Case studies of rural communities in both the US and Canada have found the water in many home wells is unsafe due to chemical and/or microbial contamination, documenting the seriousness and urgency of this public health issue [5,14,26,27,28,29,30,31,32,33,34,35,36]. A US Geological Survey (USGS) nationwide study to assess home well water quality found that 23% of 1389 wells tested across 48 states had one or more inorganic contaminant(s) at concentration(s) exceeding human-health benchmarks, primarily uranium (U), radon (Rn), manganese (Mn), nitrate (NO_3_^−^), arsenic (As) and several other trace elements [23]. Nationally, this means about 11 million people are at risk from exposure to inorganic contaminants through consumption of their home well water. 

The health risks from exposure to these contaminants are well documented and especially serious for children, infants and fetuses. Uranium increases cancer risk and leads to liver damage [37] and kidney damage [38,39]. Stored primarily in bone [40], U is associated with diminished bone growth, DNA damage, effects on the brain and other developmental and reproductive effects [41,42]. Nationally, people with the highest quartile of U in their urine had an odds ratio of diabetes of 1.46 (95% CI 1.09–1.96), compared to people in the lowest quartile of urine U [43]. A recent review of health effects is available in the Toxicological Profile for Uranium [44].

Manganese concentrations ≥0.30 mg/L in drinking water may cause neurologic damage in children, including lowered IQ, learning disabilities and changes in behavior and memory [45,46,47,48,49,50,51,52,53,54,55]. Exposure as short as ten days can be damaging to human infants [56] and pre-natal exposures are associated with higher rates of infant mortality [57]. Oral exposure, in addition to causing detrimental neurological effects in adults (manganism), could be affecting multiple aspects of male reproductive function [58]. The health risks have been well summarized [59], and are sufficiently serious for the EPA to have issued a Health Advisory and the USGS to have set a Health Based Screening Level for manganese in drinking water of 0.30 mg/L [56,60]. 

Nitrate levels exceeding 10.0 mg/L are of concern, especially for pregnant women, infants and children. Fetal exposure to elevated NO_3_^−^ has been associated with higher risk of intrauterine growth retardation, sudden infant death syndrome, cardiac and nervous system defects [61], and spina bifida, limb deficiency and other physical defects [62]. Infants exposed through their drinking water or formula are at risk for methemoglobinemia, a potentially life-threatening condition [61]. Exposure to drinking water NO_3_^−^ levels below the maximum contaminant level (MCL) has been repeatedly correlated with increased risk of developing Type I childhood diabetes [63]. In adults, chronic exposure can cause spleen hemorrhaging [61]. There is conflicting evidence of increased risks of leukemia, brain tumors and nose and throat tumors in children, and/or risks of stomach, gastric and other cancers in adults [61]. A comprehensive review of drinking water NO_3_^−^ health effects is provided by Ward et al. [63].

Arsenic exposure through consumption of contaminated drinking water is a global public health issue, including in the United States [64]. It is known to increase risks of skin, kidney, bladder, lung and liver cancer [65,66]. Arsenic acts as an endocrine disruptor, even at low concentrations [67], and affects immunological function, increasing risk for infection and autoimmune disease [68,69,70]. Exposure is associated with diabetes [71], with intellectual impairment in children consuming well water with >0.005 mg/L As [72] and with other health effects on almost every organ of the body [73]. 

The seriousness of the risks from consuming contaminated well water was recognized by the American Academy of Pediatrics in a policy statement entitled “Drinking Water from Private Wells and Risks to Children.” This statement reviews health effects of common contaminants, recommends an approach to well water testing, publicizes relevant on-line resources and encourages states and counties to subsidize the cost of testing for families who cannot afford it [24,74].

Despite these known health risks, studies show that most people do not have their well water tested [75,76]. Factors contributing to the low testing rate include lack of knowledge, lack of time, cost, privacy concerns, inconvenience, inability to understand test results and the common misperception that if water tastes and smells fine it must be safe to drink [30,33,77].

Further, data on well water treatment and consumption are scarce: neither the USGS study [23] nor the vast majority of other cited studies collected such data. The USGS study acknowledged that consumption data matched to well water quality data are needed and essential to assessing and mitigating the human health risks from contaminated home wells [23]. Research that includes an assessment of people’s consumption of unsafe well water, such as the study reported here, will also be vital for designing and implementing appropriate public health action [20,22].

Additionally, well water contamination research would benefit from utilizing a cumulative risk assessment methodology, as illustrated by the USGS study finding that 73% of tested wells “had two or more contaminants greater than one-tenth of their individual benchmarks” [23]. In that study, cumulative risk assessment methodology was not applied due to inadequate mixture specific health effects data [23]. When the health effects of chemical mixtures are poorly understood, and diverse target organs of toxicity are involved, the EPA recommends that the “hazard index” (HI) be calculated as a measure of cumulative risk [78,79]. The HI is calculated for each well by summing “the ratios of the expected exposure (E) to the chemical divided by an acceptable level (AL) for exposure to that chemical” [80] (Equation (1)):

(1) $$\mathrm{HI}=\;\overset{n}{\underset{i=1}{\sum }}\left(E_{i}\right)/\left({\mathrm{AL}}_{i}\right)$$

where *i* indicates one of the *n* chemicals.

If HI exceeds 1.0, the cumulative risk is a health concern. This approach makes it possible to conduct risk assessments of exposure to a mixture of chemicals in drinking water, albeit with the understanding that it does not address antagonistic or synergistic effects.

As the EPA emphasized in their guidance, the “Framework for Cumulative Risk Assessment,” other stress factors that affect health—such as economics and cultural traditions—*must also be considered*, in addition to physical, chemical, biological and radiological stressors, even though the methodology for taking other stressors into account was not available at the time [78,81]. Environmental justice literature subsequently highlighted how environmental contaminants, psychosocial and economic stressors, and limited access to services and resources interact to create environmental health disparities [82,83,84,85,86]. The National Institute on Environmental Health Sciences’ current five-year plan includes a goal of understanding how nonchemical stressors interact with exposures to impact health [87]. Most relevant to this analysis, Balazs and Ray [1] developed a drinking water disparities framework which includes the natural, built and sociopolitical environments, interacting at regional, community and household levels, to “explain environmental injustice in the context of [municipal] drinking water” in the San Joaquin Valley in California. Addressing complex environmental health problems is challenging and requires “interweaving multiple knowledge resources,” including community expertise [88]. The use of community based participatory research (CBPR) by tribes/minority communities and their academic partners to bring such a comprehensive approach to addressing public environmental health issues is well documented [88,89,90,91,92,93,94,95,96,97]. This approach provides a mechanism to begin to create more integrated datasets, including community expertise.

In this article, we describe our work to reduce local health risks by conducting and sharing the results of a *community-engaged*, *cumulative* risk assessment of exposure to well water contaminants, utilizing and integrating multiple knowledge sources. Quantitative and spatial data on home well water inorganic contamination, along with well water treatment and consumption data, are presented. Tribal members’ knowledge of well and septic maintenance procedures, measures to protect wells and strategies for coping with poor quality well water are discussed, based on surveys [98,99] and interviews [100,101]. Secondary health and economic data are incorporated. Balazs and Ray’s drinking water disparities framework of “natural environment” and “community and household factors” [1] proved useful for describing home well water disparities on the Crow Reservation. 

This project found that the water in 39.2% of wells is unsafe due to non-carcinogenic health risks from U, Mn, NO_3_^−^, Zn and/or As, with at least an additional 8.2% of wells safe with regards to these contaminants, but having carcinogenic risks from arsenic; despite widespread poor taste and odor, 80% of families consume their well water. Limited financial resources to treat well water, lack of environmental health literacy about well water safety, pre-existing health conditions and limited environmental enforcement contribute to vulnerability. Ensuring access to safe drinking water is a public environmental health priority for Crow and other rural US families with limited financial and environmental health knowledge resources.

### Study Area

The Apsaálooke (Crow) live in south-central Montana, on a reservation centered in the Tribe’s original homelands. About 7900 of the Tribe’s approximate enrollment of 11,000 people live on the reservation [102], maintaining the Apsaálooke language, many ceremonial practices, and relationships to the rivers, springs and other natural resources. Even today water is a respected resource essential to many prayers and other traditional practices [103,104,105].

The Apsaálooke contend with multiple health disparities [106] which increase vulnerability to environmental contamination [81]. The Crow Tribal community considers diabetes to be a top health disparity [107]; the prevalence rate in the County is 12.1%, nearly double the statewide 6.2% rate [106]. In-patient admittance for diabetes in the County at 2275/100,000 people is 2.76 times the state-wide average [108]. These disparities are more completely described elsewhere [98,99,106,109,110].

Traditionally, Tribal families hauled free water from rivers and springs for home use, and most rural residents continued to do so until the 1960s when indoor plumbing was finally installed. Subsequently, community members became increasingly concerned that consumption of poor quality home well water was contributing to illnesses in the community. After conducting a week-long reservation-wide environmental health assessment [111,112], Tribal stakeholders along with faculty from Little Big Horn College (LBHC) and Montana State University Bozeman (MSU), formed the Crow Environmental Health Steering Committee (CEHSC) in 2005 to address health risks from contaminated surface and groundwater [113]. This decade-long collaboration, operating as the CEHSC with academic partners from LBHC and MSU [94,99,105,110,114,115,116], follows the principles of community-based participatory research (CBPR) [117,118]. In keeping with CBPR principles, Tribal stakeholders, academic partners (both Crow and non-Native) and Crow college students worked and continue to work together in every step of the process from identifying the issues to be researched and mitigated, to co-authoring publications [103].

## 2. Materials and Methods 

### 2.1. Institutional Review Board Approval

The CEHSC sought and obtained Tribal approval to conduct this research through a Memorandum of Agreement signed by the Crow Tribal Chairman and the Presidents of LBHC and of MSU, followed by MSU Institutional Review Board (IRB) approvals in 2007 (#TF080107-1FC) and 2012 (#AC042012), and in 2014 by LBHC once they had established an IRB (#2014-12-01). Survey questions were adapted from earlier water ingestion research [119], then the adapted questionnaire was reviewed and refined by two Crow CEHSC members with relevant Masters degrees. Survey questions covered treatment and uses of home well water; well water color, odor and taste; other sources of drinking and cooking water; well and septic system knowledge and maintenance practices; potential sources of well water contamination and other factors. Crow Tribal Project staff and CEHSC members recruited participants, with a concerted effort to adequately represent all districts of the reservation, utilizing networks of friends and family; flyers and local papers; tables at health fairs, Tribal offices and other local venues; and snowball sampling. The local Project Coordinator, a Tribal member, met with each respondent at their home, explained the project, answered questions, and discussed and completed the consent form and survey with them. The survey methods are more completely described in Eggers et al. [99]. 

### 2.2. Sample Collection and Analysis

Well water samples were collected for microbial and chemical analyses from participants’ kitchen sinks. The tap was run for several minutes until the water temperature had stabilized, then temperature, conductivity and pH were measured with a multimeter (Oakton, Vernon Hills, IL, USA). Three sample bottles provided by a USEPA certified analytical lab were used to collect samples for inorganics, nitrate, and coliform and *E. coli* analyses, respectively, following sterile procedure. Samples were immediately placed on ice and hand-delivered within eight hours to the EPA certified lab in Billings, Montana, following chain of custody procedures [120]. The lab ran a “complete domestic analysis” on each sample (Table 1), including metals (aluminum, arsenic, cadmium, calcium, chromium, iron, lead, magnesium, manganese, potassium, sodium and zinc), inorganics (alkalinity, bicarbonate, carbonate, chloride, sulfate, fluoride, nitrate + nitrite as N, hardness as CaCO_3_ and sodium absorption ratio), physical properties (conductivity, corrosivity, pH, and total dissolved solids) and coliform/*E. coli* presence/absence. Uranium was not initially included as it was not part of the lab’s “complete domestic analysis,” and historically had not been tested for in Montana’s wells [121].

After sampling about 50 wells, testing for cadmium, chromium and lead was discontinued, as detections were infrequent and far below drinking water standards. Additionally, the proper sampling protocol for lead proved difficult to implement with participants. These funds were reallocated to testing for U, based on local geology [123,124]. A more complete description of sample collection and analytical methods, as well as U test results, is provided in Eggers et al. [99].

As seasonal fluctuation in concentrations of inorganic well water contaminants was assumed to be limited, each well was only tested once to maximize the number of families served. Based on 2010 US Census Bureau data [125], USGS data [23] and project data, it was estimated that 1020 Crow families relied on home wells, so a minimum goal of 100 participating families was set. One hundred ninety-seven people from 165 Tribal families completed surveys and 164 wells were tested.

### 2.3. Data Management

ArcMap 10.4.1 (ESRI, Redlands, CA, USA) was used to create a geographic information system map of well water contaminants. A random perturbation geomasking method, known as the donut method, was employed to protect participant confidentiality for all cartographic representations [126,127,128,129]. (See Supplementary Materials). All cartographic products were reviewed by the CEHSC for confidentiality. Contaminant concentrations were log transformed (into log base 10) and pair-wise (e.g., log[NO_3_^−^] and log[U]) and multiple (e.g., log[Fe], log[Mn] and log[TDS]) correlations were analyzed using regression in IBM SPSS™ Statistics 22 (IBM Corporation, Armonk, NY, USA).

### 2.4. Well Water Contamination Assessment

The concentrations of inorganic contaminants in each well were compared to the respective EPA Maximum Contaminant Level (MCL), Health Advisory (HA) level and/or Secondary Maximum Contaminant Level (SMCL). As nitrate plus nitrite were measured as nitrogen (hereinafter “nitrate” or “NO_3_^−^”), the corresponding MCL of 10.0 mg/L was used. There is no MCL for Mn, so the EPA HA for Mn was used. For each inorganic contaminant with an MCL or HA, for each well, Risk Quotients (RQs) were calculated by dividing exposure by the endpoint, following Barber et al. [130] (Equation (2)):

RQ = Exposure/Endpoint(2)

where exposure is the well water contaminant concentration (in mg/L), and the endpoint is the EPA drinking water MCL or HA (in mg/L) for that contaminant. A well with any single RQ exceeding 1.0 indicates it does not meet EPA safe drinking water standards for public supplies. The percentages of wells exceeding an RQ of 1.0 for each of the contaminants were then calculated. 

As a measure of overall water quality, a Sum of RQs value was calculated for each well. To assess the spatial distribution of poor well water quality, the percent of wells in each Reservation ZIP code (postal code) with summed RQ values exceeding 1.0 was calculated, along with the average and standard error of each ZIP code’s summed RQ values. These calculations were based on Mn, U, As and NO_3_^−^ only, as there is no MCL for Zn, and as testing for Cd, Cr and Pb had been discontinued due to infrequent, low level detections.

GIS maps of well water RQ values for NO_3_^−^, Mn and U were generated and are provided in the Supplementary Material (NO_3_^−^ and Mn) or were previously published (U) [99]. The percentages of wells testing positive for coliform and for *E. coli* contamination were also calculated and mapped using GIS (percentages reported, but map not shown).

### 2.5. Cumulative Risk Assessment

#### 2.5.1. Non-Carcinogen Risk: Hazard Indices

The cumulative non-carcinogenic health risk from U, Mn, As, Zn and NO_3_^−^ exposure for each well was assessed by calculating the Hazard Index (HI) [80], as described in Equation (1).

Exposure (E) to each contaminant was determined by calculating the Average Daily Dose (ADD) in mg/kg-day following the EPA’s Guidelines for Exposure Assessment [131], as shown in Equation (3):

ADD = [C × IR × ED]/[BW] × [AT],(3)

where C is the average concentration of the contaminant (mg/L), IR is the average intake rate (L/day), ED is the exposure duration (days), BW is body weight (kg), and AT is the time period over which the dose is averaged (days) [131]. 

Participants reported drinking an average of eight cups of well water per day, so the EPA’s assumed daily water consumption of two liters/day for adults was used for the IR parameter [56]. Study participants were not asked to report their body weight because the CEHSC deemed that information too personal and thought asking for that information would discourage participation. All participants were adults, hence the EPA’s assumed body weight of 70 kg was used for the BW parameter [56]. Rural Crow Tribal families typically pass homes down from generation to generation so the exposure duration (ED) and the averaging time (AT) was assumed to be a lifetime: 70 years or 25,550 days [132,133]. (As a simplification, IR and BW were not adjusted for years spent in childhood.).

The Acceptable Levels (AL) in Equation (1) for non-carcinogenic health risks are the Reference Doses (RfDs) (Table 2). 

RfD values are derived by dividing the No Observed Adverse Effects Level (NOAEL) by uncertainty factors (UF) and modifying factors (MF), as shown in Equation (4) [132]:

RfD = NOAEL/[UF × MF]. (4)

Most of these RfD values (Table 2) are from the EPA’s Integrated Risk Information System (IRIS) [128]. Uranium is a notable exception. The EPA IRIS webpage for uranium currently gives the Oral RfD as “not evaluated” [134]. However, the EPA’s Office of Superfund Remediation and Technology Innovation (OSRTI) issued a formal memorandum in 2016 stating that the EPA IRIS RfD from 1989 is not based on current science, as acknowledged in 2002 by the National Center for Environmental Assessment (which oversees IRIS). OSRTI recommended using the Agency for Toxic Substances and Disease Registry’s Minimal Risk Level of 0.0002 mg U/kg-day as the RfD for risk assessments from chronic exposures [137].

Next, the Hazard Quotient (HQ) for each contaminant for each well was calculated to determine the non-carcinogen risk, by dividing the ADD by the respective oral RfD (Equation (5)) [133]:

HQ = ADD/RfD.(5)

Per Equation (1), the HQs for each contaminant at each well are summed to determine the well’s Hazard Index (HI). An HI value greater than 1.0 for a home well indicates there are non-carcinogenic health risks of concern from consuming that water over a lifetime. 

#### 2.5.2. Carcinogenic Risk: Arsenic and Uranium

Arsenic and uranium are known carcinogens so risk of cancer was also evaluated. The carcinogenic risk from arsenic exposure through drinking water can be calculated by multiplying the ADD (Equation (3)) by EPA IRIS’s oral slope factor for arsenic of 1.5 mg/kg-day [133,138]. Determining the acceptable level of cancer risk for As in water is challenging. A de minimus cancer risk value of 10^−6^, representing a cancer development risk less than 1 person in 1,000,000, is broadly used in federal environmental programs, including the Clean Water Act, the Clean Air Act, and the Comprehensive Environmental Response, Compensation and Liability Act [139]. However, assessing cancer risk as low as 10^−6^, from consuming two L/d of As-contaminated water over a lifetime, would require the (unavailable) technical ability to detect As down to 4 × 10^−6^ mg/L, based on Equations (1), (3) and (5). EPA 2000 guidance for States and Tribes recommends that risk values be set at 10^−6^ or 10^−5^ [139]. At the As detection limit of this study, 1 × 10^−3^ mg/L, the lifetime cancer risk from consuming two liters of water per day would be 4.3 × 10^−5^, based on Equations (1), (3) and (5). Hence, 4.3 × 10^−5^ was set as the carcinogenic risk level from arsenic for this study—above 10^−5^, but the lowest allowed by the available technology. By way of comparison, the cancer risk of the 0.010 mg/L MCL for As is quite high: 2.5 × 10^−3^ [140], reflecting the necessity of considering technical feasibility and costs for public water supplies. 

EPA’s Integrated Risk Information System (EPA IRIS) has not set an oral slope factor for uranium [141]. The EPA’s Hierarchy of Human Health Toxicity Value Sources provides tier two and tier three toxicity value sources when IRIS values (tier one) are not available [142]. The highest ranked toxicity value for uranium carcinogenicity found is provided by the EPA’s Health Effects Summary Tables (HEAST) [143]. This document provides a Slope Factor for lifetime excess total cancer risk per unit intake via water ingestion of 6.40 × 10^−11^ (risk/picocurie) for U-238, and of 7.07 × 10^−11^ for U-234 [143]. As this project analyzed total uranium in water in mg/L (for comparison to the U MCL), mass of U had to be converted to activity in pCi/L. There is no single conversion factor for this, as chronic exposure to two of uranium’s radioactive isotopes, U-234 and U-238, increases risk of carcinogenic health effects in humans, and the ratio of these two isotopes varies with the water supply [144]. U-234 releases more alpha radiation per unit mass, but U-238 represents most of the mass [144]. Hence, EPA conversion factors for U mass to activity range from 0.67 to 1.5 pCi/µg [145]. Lacking data on the U-234/U-238 ratio in each well, this analysis applied the activity-to-mass ratio used by the EPA to set the U MCL: 0.9 pCi/µg [145] (Equation (6)):

U ADD (mg/kg-day) × 1000 µg/mg × 0.9 pCi/µg = U ADD (pCi/kg-day) (6)

As U-238 represents most of the mass, 6.40 × 10^−11^ (risk/picocurie) was used as the Slope Factor. (Using a weighted average for the Slope Factor would not have changed the outcome of the assessment.) (Equation (7)):

U ADD (pCi/kg-day) × 6.40 × 10^−11^ (risk/pCi) = lifetime excess total cancer risk. (7)

Acceptable values for lifetime excess total cancer risk from U exposure were considered to be less than 1 × 10^−6^.

### 2.6. Indian Health Service Well Water Data 

The local Indian Health Service Hospital (IHS) provided paper records on about 650 wells drilled for Tribal members on the reservation since the 1960s; these records provided water quality test results at the time the well was drilled. However, IHS rarely tested for As, almost never tested for U, and until recently did not record well locations by latitude and longitude. While these data were therefore insufficient for HI calculations and GIS mapping, the data were useful for assessing whether this project over-, under- or adequately sampled wells with high TDS, Mn and/or nitrate.

For each analyte, the percent of wells in both the project and the IHS databases that exceeded the EPA primary and/or secondary standard were calculated using MS Excel™ 2010 (Microsoft Corporation, Redmond, WA, USA). The comparable percentages for wells nationwide were obtained from the USGS [23]. 

### 2.7. Survey Data

Survey data were entered into MS Access™ 2010 (Microsoft Corporation, Redmond, WA, USA) and subsequently analyzed using IBM SPSS™ Statistics 22 (SPSS Inc., Chicago, IL, USA). Comparisons of categorical variables were conducted using chi square or ANOVA in SPSS. When there was significantly unequal variance between contaminant concentration distributions, Dunnett’s non-parametric test was used instead of ANOVA.

### 2.8. Interview Data

Economic, cultural and behavioral factors related to water contamination were elucidated via in depth interviews with 30 key informants representing all Districts of the Crow Reservation. Messengers for Health, a local non-profit, collaborated on this effort and IRB approval was obtained from MSU (#DK100814 modification), as there was no Crow Tribal or LBHC IRB at the time. After signing consent forms, informants interviewed with a Crow CEHSC member or Crow graduate student. Interviews were transcribed and analyzed by CEHSC members using content analysis. The transcripts were initially examined to determine which themes related to home well owners’ perceptions of their water and were the most common throughout the interview process. Interviews were then reviewed and coded; discrepancies were reviewed and resolved by an in-depth discussion. A master list of themes was recorded once a consensus was reached. Codes were applied to all interviews and a sample of 10% was validated by the first author with an interrater reliability score of 70%. The financial hardships associated with contaminated well water are described in this article; a more comprehensive analysis has been presented [100] and is pending publication [101].

### 2.9. Risk Communication and Mitigation

Risk communication and mitigation were also vital project components. Each participant was provided with an individualized letter explaining any associated health risk and/or aesthetic or plumbing issues as well as mitigation options, the results from the EPA certified lab, a spreadsheet comparing their well water contaminant concentrations with EPA’s primary and secondary standards and highlighting exceedances, and one-page handouts on each contaminant of concern. A publication entitled “Household Drinking Water Protection and Treatment” [146] as well as a DVD, “Taking Care of Your Ground Water: A Homeowner’s Guide to Well and Septic Systems,” [147] were also included. This DVD included a demonstration and explanation of how to shock chlorinate one’s well. Subsequently, project staff contacted and visited with as many participants as possible to explain and discuss the results and mitigation options for both inorganic and coliform contamination. 

Project results were widely presented to the community at meetings of the CEHSC, the Crow Tribe’s Water Resources office, the Pryor 107 Elder’s Committee, Messengers for Health, and the community at large. Data were also provided to the Crow Tribal Chairman, the Crow Tribal Environmental Protection Department, the Environmental Health Department at local Indian Health Service Hospital, and the Apsaalooke [Crow] Water and Wastewater Authority (AWWA). In the schools, outreach efforts targeted Reservation K-12 teachers and middle school children and their parents. Final results were submitted to the tribal newspaper for publication. While the funding source allowed dissemination efforts, collecting data on the effectiveness of community education as an “intervention” was not permitted (see Section 4.7 Future Research).

## 3. Results

### 3.1. Natural Environment

#### 3.1.1. Inorganic Well Water Contaminants Exceeding EPA Primary Drinking Water Standards

The mean, standard deviation, minimum, maximum and percent detection values for each analyte were calculated (Table 3). Half the value of the reporting limit was substituted for non-detect values, except for Cd and Cr, which were rarely detected. As it proved impractical to collect water samples accurately reflecting Pb concentrations from both groundwater and pipes, and only several homes had corrosive water (all from the incorrect use of a water softener), the limited Pb data are not shown.

Four inorganic contaminants—U, Mn, NO_3_^−^ and As—occurred widely in the home well water of rural Crow Reservation families, with 23.4% of wells exceeding EPA primary drinking water standards [148] based on these four constituents. Eleven percent of wells tested exceeded the Mn MCL, 6.2% exceeded the U MCL and 4.3% the NO_3_^−^ MCL; As was detected in more than a quarter of home wells although it rarely exceeded the standard (Table 4).

**Figure 1:**
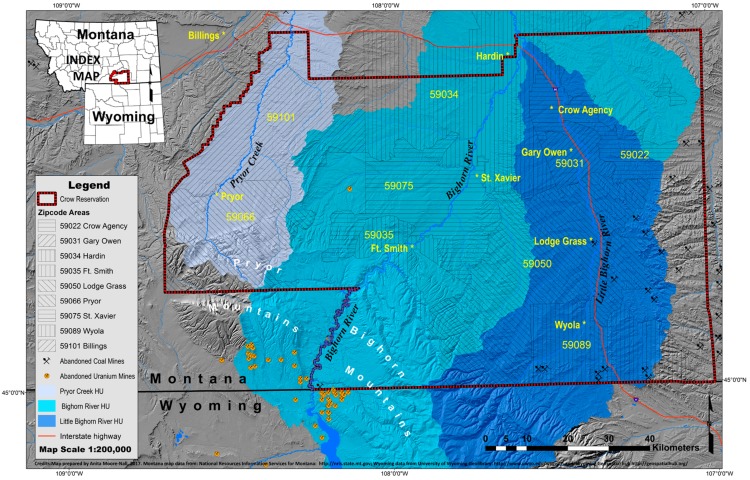
The local ZIP code regions are known to Reservation residents and map reasonably well to watersheds: Pryor and South Billings ZIP codes are in the Pryor Creek Hydrologic Unit (HU); Fort Smith, St. Xavier and Hardin fall within the Bighorn Lake and River HU; and the Wyola, Lodge Grass, Garryowen and Crow Agency ZIP codes comprise the Montana portion of the Little Bighorn River HU.

All four contaminants (U, Mn, NO_3_^−^ and As) are widely distributed in wells in the Bighorn River watershed (hydrologic unit). This watershed had the worst well water quality, with the sum of Risk Quotients (RQs) exceeding 1.0 in 64.3% of wells, and an average sum of RQs of 2.16 (Table 5). In the Little Bighorn River valley, U, Mn and/or As contamination were found primarily in wells in the Crow Agency and Wyola ZIP codes (Figure 1); 23.1% and 28.0% of wells in these two ZIP codes had summed RQs exceeding 1.0, with average summed RQs of 1.09 and 0.84 respectively (Table 5). Most wells in the Pryor Creek watershed were safe to drink with respect to these inorganic contaminants. Reservation wide, based only on the EPA drinking water standards for Mn, U, As and NO_3_^−^, almost one in four home wells tested was unsafe for consumption, and the average sum of RQs was 0.92 (Table 5). 

Maps and ZIP code analyses of the distributions of Mn and NO_3_^−^ are provided in the Supplementary Materials (Figures S1–S4); comparable data for U, as well as a further discussion of U in relation to local geology and the history of mining, is provided in Eggers et al. [99]. 

#### 3.1.2. Inorganic Well Water Contaminants Exceeding EPA Secondary Drinking Water Standards

Every tested well (*n* = 164) exceeded one or more of the EPA primary (health) [148] and/or secondary (aesthetic, cosmetic and/or technical) standards [149] set for municipal water supplies. Eighty-six percent of wells exceeded the Secondary Maximum Contaminant Limit (SMCL) for total dissolved solids (TDS), 77% exceeded the SMCL for hardness, 69% for sulfate, 33% for Mn (≥0.05 mg/L) and 25% for Fe (Table 4). 

#### 3.1.3. Microbial Well Water Contaminants Exceeding EPA Primary Drinking Water Standards

More than 40% of Crow home wells tested positive for coliform bacteria, which indicate possible fecal contamination, although less than 1% were positive for *E. coli* [98,148]. The risk assessment method used here does not provide a way to incorporate potential microbial health risks, hence these risks are not addressed in this article. However, addressing presence of coliform bacteria in home wells was an important part of the mitigation work with homeowners.

#### 3.1.4. Inorganic Well Water Contaminants below EPA Primary Drinking Water Standards

Of 55 wells testing for Cd, none exceeded the detection limit of 0.001 mg/L (Table 3); the MCL for Cd of 0.100 mg/L is 100-fold higher than the detection limit. Of these 55 wells, Cr was detected in four at the detection limit of 0.010 mg/L, which is only 10% of the MCL (0.10 mg/L) (Table 3). These four wells were all in the Fort Smith ZIP code in the Bighorn River Valley. Zinc, at a detection limit of 0.01 mg/L, was found in 80 (48.5%) of 165 wells tested. The Zn concentration exceeded 1.0 mg/L in only two of these wells (Table 3). Based on these results, analyses of Cd and Cr were discontinued, and those funds were re-budgeted to test for uranium. There is no EPA MCL for Zn, however there is a Reference Dose; testing for Zn was continued and those data were incorporated into the risk assessment described below.

#### 3.1.5. Predicting Occurrence of Hazardous Inorganic Contaminants

Although not all wells with high TDS have unsafe levels of inorganic contaminants, higher TDS was associated with manganese as well as with uranium contamination (Table 6). Regression analyses found that log[TDS] and log[NO_3_^−^] were positively correlated with log[U] (*p* < 0.0005) (see also [150]); and that log[TDS] and log[Fe] were positively correlated with log[Mn] (*p* < 0.0005). pH was negatively correlated with log[U] and log[Mn] (Table 6). Neither [As] nor [NO_3_^−^] were significantly correlated with [TDS].

Project staff have found these correlations useful for discussing potential risks with well owners who are considering whether to have their well water tested. (Data on the effectiveness of the project’s community health education is pending, as our existing funding cannot be spent on assessment of interventions. See Section 4.7 Future Research.)

#### 3.1.6. Cumulative Risk Assessment: Non-Carcinogenic Risk

The calculation of Hazard Quotients resulted in 34 wells being identified as unsafe for lifetime consumption (HQ > 1.0), two of them for arsenic and 32 of them for uranium, representing 1.2% and 33.0% of wells tested for that contaminant, respectively (Table 7). No well had an HQ exceeding 1.0 for NO_3_^−^, Mn or Zn, despite seven wells having NO_3_^−^ concentrations exceeding the EPA MCL and 18 wells having Mn concentrations exceeding the EPA HA, representing 4.3% and 11.0% of wells tested, respectively (Table 7).

Summing the HQs for each well resulted in Hazard Indices (HIs) for 38 wells exceeding 1.0, i.e., unsafe due to non-carcinogenic risks. This represents 39.2% of the 97 wells for which complete data (NO_3_^−^, As, Mn, Zn and U concentrations) were available. The average HI for these wells was 1.40, substantially above the 1.0 limit (Table 8). This compares to only 23 wells, or 23.7% of wells, being identified by summing Risk Quotients, as having sufficiently poor water quality to be of health concern (Table 8).

**Figure 2:**
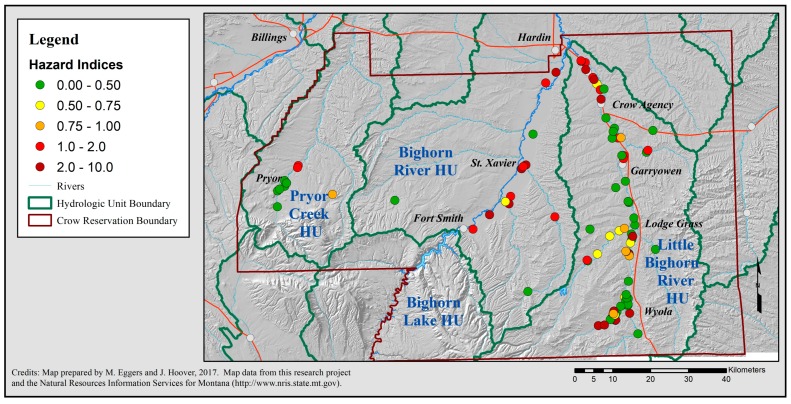
Hazard indices for home wells on the Crow Reservation. Values 1.0 and higher (red and dark red circles) represent wells with water unsafe for lifetime consumption. Town locations, rivers and boundaries provided by States of Montana and Wyoming [151,152].

Mapping the HIs shows an overall spatial pattern very similar to the distribution of the Summed RQ values from the above water quality analysis: The Bighorn River Valley and the Crow Agency and Wyola ZIP codes of the Little Big Horn River Valley have the most wells with water unsafe for consumption (Figure 2). The locations of wells have been geomasked, i.e., the locations have been shifted slightly to protect the identities of the well owners, while preserving the overall spatial distribution pattern [126].

#### 3.1.7. Cumulative Risk Assessment: Carcinogenic Risk 

As this project’s reporting limit for As was 1.0 × 10^−3^ mg/L, every well where As was detected had increased risk of cancer in excess of one person in a million, with values ranging from 4.29 × 10^−5^ up to 1.20 × 10^−3^ (Table 9). No well tested had an additional carcinogenic risk from uranium of 1.0 × 10^−6^ or more, with the maximum added risk being 1.7 × 10^−10^ in one home well (Table 9).

#### 3.1.8. Cumulative Risk Assessment: Non-Carcinogenic Plus Carcinogenic Risk

Calculation of Hazard Indices resulted in identifying 39.2% of wells (38/97) as having unsafe water due to non-carcinogenic risks. Additionally, 26.8% of wells (44/164) were found to have water unsafe due to carcinogenic risks from arsenic, based on an increased risk of cancer of ≥4 × 10^−5^. Of the 97 wells assessed for non-carcinogenic risk, 8.2% (8 wells) had an HI < 1.0 but detectable As. Hence, the risk assessment method found that 39.2 + 8.2% = 47.4% of wells have water which is unsafe for lifetime consumption due to inorganic contaminants, compared to the water quality assessment which found 23.7% of wells to have unsafe water. 

### 3.2. Community and Household Factors

#### 3.2.1. Well Water Consumption, TDS and HI

Family well consumption patterns were compared to total dissolved solids (TDS), as a measure of palatability, and HI values as a measure of (non-carcinogenic) health risks (Table 10). The EPA SMCL for TDS is 500 mg/L, i.e., TDS levels above 500 mg/L are undesirable for consumption. Based on survey data, 60% of rural Crow families drink and cook with their well water; the average TDS in these wells, at 959 ± 578 mg/L, is nearly double the SMCL (Table 10). Another 20% of families generally do not drink the water, but still cook with it; the average TDS of their well water is significantly higher (*p* = 0.002), averaging 1970 ± 1466 mg/L (Table 10). The remaining 20% of families neither drinks nor cooks with their well water; their average well water TDS is 2262 ± 1726 mg/L, more than quadruple the SMCL, and significantly higher than well water TDS in the first group (*p* = 0.001) (Table 10). 

Decreased well water quality as measured by TDS corresponded to increased risk that the water is unsafe for consumption due to non-carcinogenic risks from inorganic contaminants. Of those families who consider their well water acceptable for both drinking and cooking, (average TDS 959 ± 578 mg/L), 26.4% have unsafe level(s) of Mn, U, As, Zn and/or NO_3_^−^, based on HI values (Table 10). An additional 7.3% (4 out of 55) wells had an HI below 1.0 but added carcinogenic risk from arsenic. Among families who cook with water too unpalatable for drinking, (average TDS 1970 ± 1466 mg/L), nearly 43% are being exposed to an unsafe combination of inorganic contaminants as calculated by the hazard index (Table 10), with the caveat that their risk may be reduced if they consume less than two liters per day in foods including soups and stews. An additional 9.5% of wells in this group (2 of 21) had an HI < 1.0, but had added carcinogenic risk from arsenic. Families who neither drink nor cook with their well water have the highest average TDS, and almost two thirds of these wells have unsafe water, considering just HI values for non-carcinogenic risks (Table 10). 

Based on the above consumption statistics, across the reservation about 24.5% of the roughly 1020 Crow families with home wells (250 families) are exposed to unsafe levels of hazardous inorganic contaminants because they *do* consume their well water. Project survey data documented an average of 4.41 people/household, so about 1100 people are at risk for non-carcinogenic health effects. At least 26.8% of 1020 families, or about 1200+ people, had detectable arsenic in their well water and hence are at slightly increased risk of cancer. Another 20% (about 220 families, 970 people) have such unpalatable well water that they haul all water for drinking and cooking.

#### 3.2.2. Coping with Poor Quality Well Water

Despite widespread poor well water quality, almost no one uses water treatment technology. Comparing water quality data with survey data shows that although 77% of all wells tested have unacceptably hard water (above the EPA SMCL), only 3.3% of families had installed a water softener. While 86% of all wells tested exceed the SMCL for TDS, only 4% of families had a reverse osmosis system. Although more than a third of wells have Fe and/or Mn above the SMCL, no participating family had installed an Fe/Mn removal unit. 

Project survey data document that Crow families cope with unpalatable water by using alternate water sources, most commonly purchasing bottled water (53% of all families surveyed), with additional alternatives being filling jugs at municipal water sources (17%), and/or another family’s home (12%) and/or springs (4%).

Why do some families purchase and/or haul water for all consumptive uses, while others continue to cook with unpalatable water? A comparison of well water quality between “Do not consume” families vs. “Cook only” families found no significant difference in concentrations of contaminants that could possibly affect taste or color (*p* < 0.05), considering TDS, Mn, sodium, sulfate and presence of coliform bacteria. Nor is the difference between these two groups of families due to water odor: 71.4% of families who only cook with their well water noted their well water had an odor, compared to 87.5% of “no consumption” families; the difference was not statistically significant (*p* = 0.27, Fisher’s 2-sided test). 

Although the well water of “Do not consume” families had higher iron on average (1.33 ± 2.15 mg/L) than “Cook only” families’ well water (0.58 ± 1.09 mg/L), the difference did not quite reach the level of significance (log[Fe], *p* = 0.054). The SMCL for iron is 0.30 mg/L, so the average iron concentration at which families do not drink but still cook with their water is almost double this standard. Among families who haul water for all consumptive uses, the average iron concentration is four times the SMCL. 

The in-depth interviews documented that families who can afford commercial bottled water usually buy it in cases from stores in larger towns 20 to 65 miles away [153]. For some families, the cost of bottled water is an economic hardship [100,101,153], so they continue to use their well water for cooking. One mother commented that both her daughter and husband would get upset stomachs from drinking their water, but that they couldn’t afford enough bottled water for both drinking and cooking, adding, “*On occasions like holidays and stuff, I even will buy extra water, so I cook with that so that my husband doesn’t get sick or my daughter doesn’t get sick.*” 

#### 3.2.3. Community Well Stewardship

Survey results showed a widespread lack of environmental health literacy about well stewardship. Only 21% felt confident they understood well function, maintenance and safety procedures. However, this group was no more likely (*p* < 0.05) to have a sanitary (waterproof) cap on their well head (Fisher 1-sided, *p* = 0.058), to have pumped their septic system in the past two years, nor to be keeping livestock away from their wellhead—measures which are known to help protect well water from fecal contamination and potential presence of pathogens [147,154,155]. Of other survey respondents, 20% did not answer the question about their home well knowledge, and 58% thought they would benefit from learning more (112/192 people). Of those 112 respondents, 55% would like information mailed to their home, 36% were interested in having someone visit to explain in person, 27% thought articles in local papers would be helpful and only 22% would consider attending a public information session offered in their community. (In response to a query about other suitable avenues for public education, no one suggested social media, although if those venues had been listed as options there might have been positive responses.) 

While other studies have found low rates of home well water testing [30,33], not a single well owner surveyed had ever arranged to have their well water tested, prior to participation in this project. A handful of people remembered whether the Indian Health Service (IHS) had told them their water was safe or unsafe at the time it was drilled, but no one had a copy of their original well test results.

## 4. Discussion

### 4.1. Natural Environment

Water quality testing showed that 23.7% of wells sampled have unsafe drinking water based on their summed RQs ≥ 1.0, calculated from concentrations of Mn, U, As and NO_3_^−^. This is comparable to USGS’s nationwide research, which found that 23% of 1389 U.S. home wells had one or more inorganic contaminants exceeding a human health standard (albeit based on Mn, U, As and NO_3_^−^, plus Rn, B, F and Sr) [23]. To further understand how representative and comparable these data for the Crow Reservation are to nationwide well water data, comparisons were made with IHS and USGS data, respectively, for both secondary and primary contaminants (Table 4). These comparisons show that this project did not over-represent wells with poor water quality; rather, wells with high Mn and Fe levels are under-represented. Hence, our project likely underestimated of the percentages of wells which are unsafe and/or unpalatable due to high Mn and/or Fe levels.

#### 4.1.1. Primary Contaminants

The four contaminants which most often exceeded drinking water quality standards in this study, Mn, U, NO_3_^−^ and As, are also four of the eight top contaminants of concern nationwide in the USGS well water study [23]. Comparing local to national data, home wells on the Crow Reservation are far more likely to have unsafe levels of Mn and U, but are less likely to exceed drinking water quality standards for As [23] (Table 3). Of the remaining four on the USGS list—radon, strontium, boron and fluoride—this project only tested for fluoride, which very rarely exceeding the EPA’s MCL. Limited testing for strontium has recently begun, and available regional data for boron will be investigated to see if testing is warranted. Funding limitations, logistical challenges and lack of mitigation options (for radon inhalation) precluded and still preclude testing for radon. 

#### 4.1.2. Secondary Contaminants

Overall, the water in nearly all home wells exceeds either EPA’s TDS and/or sulfate standard, while nationwide these are issues only in a small minority of homes [23]. The percentages of home wells exceeding the SMCLs for Mn and Fe are about triple the national percentages. Excessive hardness affects most wells both locally (77% of project wells) and nationally (62% [23]) (Table 2).

Although secondary contaminants are not considered to be serious health risks, they must be considered in risk assessments as they limit families’ access to safe and palatable well water. Secondary contaminants present in Crow well water can cause gastrointestinal distress and/or adversely impact taste [149,156]. Other effects are discoloration/odor problems [149], scale deposit, pipe corrosion and leakage [146] with subsequent hazardous mold growth in homes [157], all requiring the installation of costly home well water treatment [146]—prohibitively expensive for most Crow families. 

#### 4.1.3. Cumulative Risk Assessment Based on Hazard Indices and Slope Factors vs. Summed RQs

Clearly, assessing both non-carcinogenic and carcinogenic health risks resulted in a much higher percentage of the wells being identified as having water unsafe for lifetime consumption, compared to considering water quality criteria only: 47.4% vs. 23.7%. A comparable risk assessment of the USGS nationwide well water quality data [23] might similarly identify a higher percentage of wells across the US as presenting health risks.

Importantly, these two methods of assessing lifetime risks from consumption of inorganically contaminated well water do not provide comparable results for most of the individual parameters examined in this study. While HI and Sum of RQs calculations yielded similar values for arsenic at any given concentration, this was not the case for NO_3_^−^, Mn, Zn or U. Based on lifetime consumption and Equations (1), (3) and (5) above, an HQ of 1.0 would not be reached for NO_3_^−^ until the concentration reached 55.9 mg/L, or for Mn until the concentration reached 1.6 mg/L—in both cases, levels more than five times the respective MCL. In fact, when the HA for Mn was determined, an additional factor of five was applied which was not used in setting the RfD for Mn [56]. Given the severity of health risks to infants from exposure to nitrate at levels about the MCL (of 10.0 mg/L) [61,62,63] and to Mn above the HA (of 0.3 mg/L) [56], as well as health risks across the lifespan from Mn and NO_3_^−^ [45,46,47,48,49,50,51,52,53,54,55,56,57,58,59,60,61,62,63], assessing health risks based solely on Hazard Indices appears to be inadequate. At the very least, well owners and their families should be informed of both the Hazard Index for their well, as well as any individual contaminants which exceed their respective MCL/HA. 

The RfD for Zn is particularly useful as there is no MCL for this element. From the RfD, it can be calculated (using Equations (1), (3) and (5)) that a Zn concentration of 10.5 mg/L, consumed at two L/d for a lifetime, would result in an HQ of 1.0. Hence 10.5 mg/L could be a useful endpoint for alerting people should the Zn concentration in their well water exceed this concentration.

The RfD for U is more conservative than the MCL, generating an HI of 1.0 when the concentration in water reaches 0.007 mg/L (based on a lifetime consuming 2 L/d), compared to the MCL of 0.030 mg/L. In this case, the EPA’s Office of Superfund Remediation and Technology Innovation’s 2016 recommendation to use the conservative RfD was based on more recent research on the toxicology of uranium, than existed at the time the MCL was established [137]. Interestingly, in 2003 a toxicologist working with the Navajo Nation on uranium contamination and her colleagues also studied this issue and recommended that the state of New Mexico’s Ground Water Quality Bureau adopt 0.007 mg/L U for the State’s standard [158]. This project informed well owners of how their well water U level compared with both endpoints (0.007 mg/L and 0.030 mg/L), explaining that there were low level health risks when the concentration in their well water was above 0.007 mg/L, albeit still below the MCL. 

This lack of consistency among health risk benchmarks for the same contaminant, even within one country, has been previously demonstrated [159,160]. As various benchmarks may be applied in different situations, such as regulation of public drinking water supplies vs. Superfund clean-up, there are arguably valid reasons for this. However, assessors of health risks from home well water should be aware of this variability and carefully consider which benchmarks and which methodologies to employ.

### 4.2. Community and Household Factors

#### 4.2.1. Consumption Data Are Necessary for Assessing Exposures to Well Water Contaminants

Only 14% of wells tested in this project were below the SMCL for TDS, so in the absence of consumption data one might assume only a small minority of families consume their well water—whereas project surveys document that 80% of families are consuming their water. Families don’t limit their consumption to cooking, only, until TDS is almost four times the SMCL and Fe is nearly double the SMCL, on average. This raises an important point: in the absence of public health education and mitigation efforts, some families *will* regularly consume unpalatable, potentially unsafe, well water, especially when financial resources for mitigation are limited. Importantly, people are consuming well water with higher levels of Mn than the World Health Organization assumed in discontinuing its drinking water advisory for Mn [59,161]. As documented here, the families most likely to be at risk are those who cook with water too unpalatable to drink. 

#### 4.2.2. Consumption Data Are also Vital to Assessing Population Level Health Risks

While this project found 39.2% of wells to have unsafe levels of non-carcinogenic health risks from inorganic contamination; the integration of consumption data reduced that estimate to 24.5% of rural Crow families who are in fact consuming this unsafe well water. Extremely unpalatable water (averaging four times the SMCL for both TDS and Fe) does effectively discourage consumption, and as high levels of TDS and Fe are significantly associated with elevated U and Mn, respectively, some families are thereby protected from exposure. Additional research about well water use is needed, and must address both drinking and cooking as modes of consumption.

As there are no taste, visual or olfactory signals of As or nitrate contamination, families with palatable water are not exempt from risk—one quarter (26.4%) of such wells in Crow are unsafe due to non-carcinogenic risks from inorganic contaminants (with some also having carcinogenic risks from As). Project staff often had to explain the health risks to people who believed their well water didn’t need testing because it tasted “just fine”. Clearly there is a need for community health education to increase awareness of risks from well water contaminants, especially contaminants which are tasteless, colorless and odorless and are not correlated with higher TDS.

### 4.3. Sources of Contamination

In this project, combining diverse knowledge sources—including well water quality data, surveys, geological expertise, local environmental knowledge and published literature—is helping to identify potential natural and anthropogenic sources of groundwater contamination; this is essential for communities and families to determine what steps could be taken, if any, to remediate, reduce and/or prevent contamination of their well water. Several examples are provided here, to illustrate the value of diverse knowledge sources, including community expertise.

#### 4.3.1. Nitrate

While sewage, wastewater and agriculture are the primary sources of groundwater NO_3_^−^ contamination in the U.S. [162], agricultural areas have the highest groundwater NO_3_^−^ levels of any major land use [63]. Shallow wells less than 100 feet in depth are known to be more vulnerable to NO_3_^−^ contamination [163], and the average depth of the 650+ wells in the local IHS database is only 66 ± 55 feet. On the Crow Reservation, 79% of the 2.2 million acres is being farmed [164]. Irrigated agriculture is most extensive in the Bighorn River valley where wells in this watershed had significantly higher nitrate levels than in Little Bighorn River valley (*p* = 0.029) (Supplement Figure S4). *T*-tests comparing well water data with survey data found that NO_3_^−^ concentration did *not* significantly correlate (at *p* ≤ 0.05) with distance between home well and septic systems (< or >100 feet), with whether the septic system had been pumped in the past two years or with livestock presence. In short, the primary source of high NO_3_^−^ levels in well water thus appears to be farming, rather than homeowner and/or ranching practices. 

#### 4.3.2. Uranium and Arsenic

Uranium, As and other metals occur naturally in local geological formations in the headwaters of the Bighorn River, a former mining district. Natural processes, perhaps exacerbated by past mining, could result in these elements contaminating groundwater in the Bighorn River valley below [123,124,165].

Anthropogenic sources may also be contributing to both U and As groundwater contamination. Log[U] is significantly predicted by log[NO_3_^−^] (*p* < 0.0005) (Table 4), consistent with the USGS study’s finding that U and NO_3_^−^ were the two contaminants nationwide most likely to co-occur at levels exceeding respective MCLs [23]. A subsequent literature search found that in the High Plains and Central Valley aquifers of the United States, NO_3_^−^ was significantly correlated with about 78% of areas mapped by interpolation to exceed the MCL for U (*p* < 0.05), particularly in shallow groundwater [166]. These authors note that NO_3_^−^ oxidizes U(IV) to U(VI), thus increasing its mobility in groundwater. In Germany, uranium levels in groundwater have been found to be three to 17 times higher in agricultural areas, compared to adjacent forested lands [167]; the phosphate rock processed for agricultural fertilizer can contain U as a natural impurity, and U concentration in groundwater correlates strongly with NO_3_^−^ as well as with other elements from fertilizer [167]. The formation of highly soluble uranyl-nitrate could also be contributing to the mobility and hence elevated levels of U in groundwater [167]. Hence, agricultural fertilizer applied on reservation lands could be adding U to the groundwater as well as increasing its mobility. 

Arsenic could be naturally occurring, and was found in home wells along Warm Creek, the Crow name for a stream known by Tribal members to be fed by hot springs, a potential source of As. Another identified arsenic hotspot is close to and downgradient from a site where Crow elders report livestock were historically dipped in arsenical pesticides, prior to loading onto the railroad crossing the reservation. Additionally, railway As levels in soil can be a magnitude or more elevated above background levels, due to the use of arsenical compounds as herbicides and as wood preservatives for railroad ties [168,169]. The railroad has been in operation since the late 1800s. Testing for As contamination of home wells downgradient of dipping sites, close to the railroad line and along Warm Creek is a priority for future work.

In sum, combining diverse knowledge sources in researching home well water quality helps identify potential contamination sources, which is essential to public environmental health action. 

### 4.4. Assessing Vulnerability, Planning Education and Mitigating Risks

Understanding factors contributing to communities’ vulnerability to health risks from contaminant exposures is important to risk assessment and essential to planning community education and mitigation. Average per capita income across the Crow Reservation ranges from $7354 to $8130, depending on the Tribal community—a mere 33% of the Montana average and 30% of the 2010 national average [125]. Most Crow families cannot afford to install and maintain treatment equipment; a water softener, iron/manganese removal unit and reverse osmosis system would cost around $1500, plus installation and monthly chemical costs. The nearest plumbing businesses and sources of water treatment chemicals are in Billings, Montana, 60 miles or more away from most Reservation residents. The circa 5% of Crow families who have installed water treatment equipment is extremely low; a study done in Alberta, Canada, found that 59% of well owners had installed some type of water treatment, with water softeners (25.2% of wells), iron filters (17.9%) and reverse osmosis (12.6%) being the most commonly used technologies [35]. Crow families who can afford neither treatment equipment nor sufficient bottled water for all drinking and cooking [101,153] need an even lower cost solution for safe water.

Neither the Tribe nor county conducts public education on how to maintain and protect wells and septic systems, nor what the health risks could be from contaminated well water. As survey results document, even the 20% of respondents who “feel confident” in their knowledge of well and septic maintenance practices were not adequately protecting or testing their home wells. Public environmental health literacy alone may not be sufficient to ensure effective home well water stewardship, but it is an essential and necessary foundation.

Additionally, the Apsaalooke [Crow] Water and Wastewater Authority identifies inadequate environmental enforcement and the complex legal, jurisdictional and regulatory issues on reservations as contributing to drinking water disparities [11]. Similar governmental issues have been experienced by other Tribes [19]. Other minority communities have also described insufficient enforcement of environmental regulations and inadequate laws governing non-point source pollution from agriculture as factors contributing to drinking water health disparities [1,12].

Health disparities from poor quality drinking water both contribute to and are exacerbated by existing health disparities. For instance, the diabetes prevalence rate in the county where the Reservation resides is double the statewide average [106], which could mean that more people are particularly vulnerable to the nephrotoxic effects of U. Physicians at the local community health center have noticed that people with diabetes appear to be losing kidney function more rapidly than is expected [170]. In the nationwide study noted above, those with the highest quartile of U in their urine are more likely to have diabetes [43]. While the interactions between U and diabetes are not fully understood, toxicologists have recommended that people with diabetes or kidney disease not consume water with more than 0.007 mg/L U (far below the current MCL of 0.030 mg/L) [158]. Additionally, diabetes has been associated with both Mn [171] and As exposures [71,172,173,174], which this project has shown are occurring through well water consumption on the reservation.

Hence, mitigation to improve access to safe drinking and cooking water in Crow Reservation communities must not only provide home well water testing, understand consumption patterns and explain the results to families, but must also implement economically feasible safe water alternatives. Mitigation must also include well owner and community education strategies, and should be conducted in collaboration with local health care professionals, as well as Tribal leadership and agencies with environmental enforcement authority.

### 4.5. Priority Public Health Issue

When considering recognized criteria for prioritizing and addressing exposures to environmental chemical mixtures [175], home well water is a high priority public health issue. Those criteria include:

(1) *Breadth of exposure*: 15% of the US population, about 49 million people, rely on home wells;
(2) *Nature of exposure*: Rural residents consume their well water daily for years, potentially throughout their lives, beginning with pre-natal exposures;
(3) *Severity of effects*: All four inorganic contaminants most prevalent in Crow home well water—uranium, manganese, arsenic and nitrate—can have severe, lasting health effects on infants and children and can cause disease in adults. The other four most common inorganic contaminants in U.S. well water, radon, boron, fluoride and strontium, represent additional health risks [58,176,177,178].
(4) *Likelihood of interactions*: The health effects of combinations of these contaminants have not been characterized; interactions as understood in an ecologic framework are nearly entirely unknown.

### 4.6. Limitations

While this study screened for seven hazardous metals (Mn, As, U, Zn, Cd, Cr and Pb) as well as nitrate, the risks from other radionuclides and metals, pesticides, pharmaceuticals, illicit drugs and other waterborne contaminants were not addressed in this study, hence the cumulative health risks presented are almost certainly underestimated. The only radionuclide assessed was U (cf. [179]), although based on project groundwater data and other research, radon, radium and strontium are likely to co-occur [23,99,179,180]. In southwest Montana, 14.1% of wells exceeded the U MCL; including Rn, Ra and other radionuclides, 29% of wells exceeded at least one MCL [180].

The risk assessment method utilized the EPA’s RfDs, which this analysis has shown for Mn and for NO_3_^−^, are five-fold less protective than the MCLs for lifetime consumption at the standard rate of two L/d. While the RfD for As is comparable to the As MCL of 0.010 mg/L, the carcinogenic risk at that MCL is unusually high for a regulatory value: 2.5 × 10^−3^ [140], as noted above. Adding in the analysis of carcinogenic risk from arsenic somewhat compensates for this, by identifying wells with arsenic risk down to 4.3 × 10^−5^.

Further, this study did not address additional potential routes of exposure [181] to heavy metals and nitrate, such as inhalation of airborne dust, sprayed pesticides and indoor air pollutants; consumption of commercial and wild foods; cosmetics; occupational and recreational exposures and more. The extent to which other routes of exposure are considered when the EPA sets MCLs and RfDs for drinking water would help compensate for this.

Additionally, 40%+ of Crow wells tested positive for coliform bacteria, indicating risk of fecal contamination [98,149]. However, risks from pathogen exposures were not addressed in this article, as the HI method does not provide a way to incorporate them. In addition to direct effects, infections and subsequent chronic impacts on health could interact with exposures to inorganic contaminants.

Recent research supports the project’s assumption that temporal variability in groundwater contaminant levels of Mn [182], nitrate [183], U and As [184] is limited. Such temporal variability in groundwater has been seen in specific conditions, e.g., where high recharge rates impact shallow wells [184], or in the case of As, where there are distinct rainy and dry seasons [133]. Any temporal variability would be somewhat compensated for by the project’s year-round sampling throughout two years.

This manuscript provides a deterministic risk assessment, generating a single point estimate of risk for people consuming water from each well, based on standard values for body weight, ingestion rate and length of lifetime exposure. A probabilistic risk assessment (PRA), which takes into account both variability and uncertainty, would have produced an estimated range and likelihood of exposure for each well. A PRA would thus more completely characterize the risks, and hence would be more useful in protecting more vulnerable or sensitive Tribal members, such as children or those with higher water consumption rates [185].

However, this project did not have adequate data to conduct a PRA, for several reasons: (1) The Tribal Steering Committee (CEHSC) determined that asking body weight in the survey was too personal, and would significantly discourage participation; (2) while the survey data provide an estimate of consumption from drinking, the survey did not attempt to assess well water consumption in cooked foods; and (3) each well was only tested once, so variability in contaminant concentrations is unknown.

### 4.7. Future Research

Research on the impacts of climate change and of seasonality on Crow Reservation water resources, including both home well water and river water, is underway [114]. The team is researching better methods to characterize water consumption, and will look into whether de-identified body weight data for the Tribal population is available. These data will allow a future, more complete characterization of the risks from home well water consumption [185].

Additionally, the team is currently researching factors correlated with coliform and with *E. coli* contamination of home well water. New and future research will focus on culturally appropriate, effective and economically feasible interventions to reduce exposures to home well water contaminants, increase access to safe drinking water and improve environmental health literacy.

## 5. Conclusions

This study has shown that rural Crow Reservation families are at risk from exposure to hazardous inorganic contaminants through consumption of unsafe home well water, as 39.2% or more of wells have a hazard index exceeding 1.0, and an additional 8.2% have added carcinogenic risk from As greater than 4.3 × 10^−5^, for a total of 47.4%. The risk assessment methodology identifies a higher percentage of home wells with unsafe water, especially those with U, As and Zn contamination, than the water quality assessment based on MCLs, but does not flag wells with Mn and nitrate levels up to five times the respective MCLs. Future assessments of health risks from home well water should be aware of this and carefully consider which methodologies and benchmarks, or combination of methods, to employ.

While 20% of Crow families avoid potential exposures by hauling all water for consumption, 80% drink and/or cook with their well water, despite widespread poor taste and unpleasant odors. Costs of water treatment are beyond the limited financial resources of most families. Additionally, lack of community health education about well maintenance and well water safety, pre-existing health conditions such as diabetes, and limited environmental enforcement increase community vulnerability.

Rural residents throughout the United States are at risk, as USGS well water data show that 23% of home wells nationwide have unsafe levels of chemical contaminants, based on the MCLs [23], comparable to our Sum of RQs data for Crow. Other communities lacking adequate health education about well water safety and/or without the financial resources to treat their well water may be similarly exposed. Assessing the health risks will require not only a hazard index analysis of well water and consumption data, but also surveys to determine well protection and maintenance knowledge and practices, financial and knowledge resources to mitigate well water contamination, and other factors which increase vulnerability or are protective. Some of these data could be collected through national surveys such as the U.S. Census, the National Health and Nutrition Examination Survey [186] and/or the Behavioral Risk Factor Surveillance System [187]. Identifying and promoting model state laws and regulations, e.g., those requiring a complete domestic analysis of well water before a rural home sale, or California’s law which recognizes safe drinking water as a human right [188], would help protect families from unknowingly consuming unsafe well water.

We must work to ensure public environmental health education about and access to safe drinking water for rural communities; it is a priority public health issue not only for Crow families, but for rural residents throughout the United States. 

## Figures and Tables

**Table 1:** Well water parameters tested, methods used and reporting limits. For the Lab’s EPA and other certifications, as well as their quality control manual, see their website [120].

Water Quality Parameter	Method ^a^	Reporting Limit	Unit
*Metals*			
Aluminum	E200.8 ^b^	0.1	mg/L
Arsenic	E200.8	0.001	mg/L
Cadmium	E200.8	0.0001	mg/L
Calcium	E200.7 ^c^	1	mg/L
Chromium	E200.8	0.001	mg/L
Iron	E200.7	0.03	mg/L
Lead	E200.8	0.001	mg/L
Magnesium	E200.7	1	mg/L
Manganese	E200.7/E200.8 ^d^	0.01	mg/L
Potassium	E200.7	1	mg/L
Sodium	E200.7	1	mg/L
Uranium	E200.8	0.001	mg/L
Zinc	E200.7/E200.8	0.01	mg/L
***Inorganics***			
Alkalinity	A2320 B	4	mg/L
Bicarbonate	A2320 B	4	mg/L
Carbonate	A2320 B	4	mg/L
Chloride	E300.0	1	mg/L
Sulfate	E300.0	1	mg/L
Fluoride	E300.0	0.1	mg/L
Nitrate + Nitrite as N	E300.0	0.05	mg/L
Hardness as CaCO_3_	A2340 B	1	mg/L
Sodium Absorption Ratio	Calculation	0.01	
***Physical Properties***			
Conductivity	A2510 B	1	µmhos/cm
Corrosivity (Langelier Index)	A203		
pH	A4500 H B	0.1	s.u.
Total Dissolved Solids @ 180 C	A2540 C	10	mg/L
Coliform/E. coli presence/absence	A9223	presence/absence	per 100 mL

^a^ Energy Laboratories (Billings, MT, USA) follows the standardized methods established by the US EPA Standard Methods for the Examination of Water and Wastewater, the National Institute for Occupational Safety and Health (NIOSH) and the American Society for Testing and Materials [118]. ^b^ Inductively Coupled Plasma (ICP—analytical method). ^c^ Inductively Coupled Plasma—Mass Spectrometry (ICP-MS). ^d^ These samples were analyzed by both methods, ICP and ICPMS, to ensure the best quantitated value. Both methods are approved for the analysis of Mn. Data between these methods are very reproducible [122].

**Table 2:** Oral references doses and respective citation(s) for each contaminant.

Contaminant	Oral RfD (mg/kg-day)	Citation
Arsenic	3 × 10^−4^	[134]
Cadmium	5.0 × 10^−4^	[134]
Chromium	3.0 × 10^−3^	[134]
Manganese	4.6 × 10^−2^	[134,135,136]
Nitrate	1.6	[134]
Uranium	2 × 10^−4^	[137]
Zinc	0.3	[134]

**Table 3:** Statistics for well water analytes.

Analyte	*n*	Min (mg/L)	Avg conc (mg/L) ± SD (mg/L)	Max (mg/L)	Number of Detections	Percent Detection
Nitrate + Nitrite as N	164	<0.05	1.61 ± 5.13	39.8	70	42.7
As	164	<0.001	0.0012 ± 0.0025	0.011	45	27.4
Mn	164	<0.01	0.102 ± 0.215	1.35	85	51.8
Zn	164	<0.01	0.11 ± 0.01	9.15	80	48.8
U	97	<0.001	0.008 ± 0.014	0.101	66	68.0
Cd	55	<0.001	N/A	N/A	0	0
Cr	55	<0.01	N/A	0.01	4	7.3
TDS	164	238	1425 ± 1215	9180	164	100.0
Sulfate	164	<1	682 ± 765	4750	163	99.4
Iron	155	<0.05	0.71 ± 2.19	21.7	79	51.0

**Table 4:** Percentages of wells exceeding primary and secondary standards, locally and nationally. Empty cells indicate that no data were available.

Contaminant	EPA Standard [143]	Percent of Crow Wells in Use Exceeding EPA Standards ^a^ *n* = 164; *n* for U = 97; *n* for Fe = 155	Percent of Crow Wells When Drilled Exceeding EPA Standards ^b^ *n* = 470–~650	Percent of United States Wells Exceeding EPA Standards ^c^ *n* = 1725–2160
Primary Standard				
Mn ≥ 0.30 mg/L	HA	11.0	17.0	5.2
As > 0 mg/L	MCLG	27.4		
As ≥ 0.01 mg/L	MCL	1.2		6.8
U > 0 mg/L	MCLG	68.0		
U ≥ 0.030 mg/L	MCL	6.2		1.7
NO_3_^−^ ≥ 10.0 mg/L	MCL	4.3	5.0	4.4
Coliform present	MCL	40.2		
*E. coli* present	MCL	0.6		
Secondary standard				
TDS > 500 mg/L	SMCL	85.4	93.0	14.8
SO_4_^2−^ > 250 mg/L	SMCL	68.9	75.0	3.8
Mn > 0.05 mg/L	SMCL	32.9	57.5	21.3
Fe > 0.3 mg/L	SMCL	25.0	63.0	19.1
Hardness > 120 mg/L	SMCL	76.8		62.0

^a^ Crow Water Quality Project data. ^b^ Crow/N. Cheyenne Indian Health Service data, compiled by Crow Water Quality Project. ^c^ USGS data [23].

**Table 5:** Percentages of wells with Sum of RQs exceeding 1.0 for Mn, U, As and NO_3_^−^, by river valley and by ZIP codes within Little Bighorn River Valley.

River Valley, from West to East on the Crow Reservation	Number of Home Wells	Wells with Sum of RQs > 1.0: Percent ± SE	Average Sum of RQs ± SE
Pryor Creek	9	11 ± 11%	0.51 ± 0.14
Bighorn River Valley	14	64 ± 13%	2.16 ± 0.64
Little Bighorn River Valley	74	17 ± 4%	0.73 ± 0.03
Crow Agency	13	23 ± 12%	1.09 ± 0.37
Garryowen	12	8 ± 8%	0.57 ± 0.18
Lodge Grass	24	8 ± 5%	0.52 ± 0.18
Wyola	25	28 ± 9%	0.84 ± 0.19
*Total Number of Wells*	*97*		
*Reservation Wide Average*		24 ± 4%	0.92 ± 0.03

**Table 6:** Predictors of [Mn] and [U] in Crow Reservation well water.

Predictor(s)	Dependent Variable	*n*	R^2^	Regression Significance	Regression Equation
logTDS	log[Mn]	151	0.164	*p* < 0.0005	log[Mn] = −4.60 + 0.961 logTDS
log[Fe]	log[Mn]	163	0.419	*p* < 0.0005	log[Mn] = −1.08 + 0.626 log[Fe]
logTDS, log[Fe]	log[Mn]	151	0.503	log TDS: *p* < 0.0005; log[Fe]: *p* < 0.0005	log[Mn] = −3.27 + 0.707 logTDS + 0.574 log[Fe]
logTDS	log[U]	97	0.194	*p* < 0.0005	log[U] = −5.463 + 0.9525 logTDS
pH	log[U]	97	0.341	*p* < 0.0005	log[U] = 3.45 − 0.795 pH
log[NO_3_^−^]	log[U]	97	0.160	*p* < 0.0005	log[U] = −2.34 + 0.267 log[NO_3_^−^]
logTDS, pH, log[NO_3_^−^]	log[U]	97	0.579	log TDS: *p* < 0.0005; pH: *p* < 0.0005; log[NO_3_^−^]: 0.001	log[U] = −0.016 + 0.905 logTDS − 0.686 pH + 0.167 log[NO_3_^−^]

**Table 7:** Average contaminant concentrations, average daily doses (ADDs), hazard quotients (HQs) exceeding 1.0, MCLs, MCL exceedances and average risk quotients (RQs) for each of five principal inorganic contaminants in home well water on the Crow Reservation.

Well Water Contaminant	*n*	Avg ADD * (mg/L)	RfD (mg/L)	# HQ ≥ 1.0	% HQ ≥ 1.0	Avg HQ	MCL (mg/L)	# > MCL	% > MCL	Avg RQ *
Nitrate + Nitrite as N	164	0.05	1.6	0	0	0.03	10.0	7	4.3%	0.16
As	164	3 × 10^−5^	0.0003	2	1.2%	0.11	0.01	2	1.2%	0.12
Mn	164	0.03	0.046	0	0	0.06	0.3	18	11.0%	0.34
Zn	164	0.003	0.3	0	0	0.01	N/A	N/A	N/A	N/A
U	97	0.0002	0.0002	32	33.0%	1.16	0.03	6	6.30%	0.27

* When a contaminant was not detected at the reporting limit (RL), RL/2 was substituted.

**Table 8:** Comparison of summary statistics for: health risk as measured by hazard indices (HIs) vs. well water quality as measured by the sum of risk quotients (RQs).

Hazard Indices (Sum of Hazard Quotients) Based on Oral Reference Doses (RfDs)		Sum of Risk Quotients on EPA Water Quality Maximum Contaminant Levels (MCLs)	
Number of wells with HI > 1.0	38	Number of wells with summed RQ > 1.0	23
Percent of wells with HI > 1.0	39.2	Percent of wells with summed RQ > 1.0	23.7
Average HI of wells	1.40	Average summed RQs of wells	0.93

**Table 9:** Comparison of summary statistics for: health risk as measured by hazard indices (HIs) vs. well water quality as measured by the sum of risk quotients (RQs).

Carcinogenic Risk from Arsenic		Carcinogenic Risk from Uranium	
Number of wells tested for arsenic, reporting limit of 0.001 mg/L	164	Number of wells tested for uranium, reporting limit of 0.001 mg/L	97
Number of wells with detected arsenic	44	Number of wells with detected uranium	66
Percent of wells with detected arsenic	26.8	Percent of wells with detected uranium	68.8
Number of wells with carcinogenic risk ≥ 4 × 10^−5^	44	Number of wells with carcinogenic risk ≥ 1.0 × 10^−6^	0
Percent of wells with carcinogenic risk ≥ 4 × 10^−5^	26.8	Percent of wells with carcinogenic risk ≥ 1.0 × 10^−6^	0.0

**Table 10:** Well water consumption, compared to palatability as measured by TDS and to health risk as measured by HI (calculated from Mn, U, As, Zn and NO_3_^−^ concentrations).

Families’ Well Water Use	*n* (%)	TDS (mg/L) Mean ± SD	Wells Assessed for HI			
			*n*	Number with HI ≥ 1.0	Percent with HI > 1.0	Mean HI ± SD
Drink & cook	91 (59.9%)	959 ± 578	55	14	26.4	0.7 ± 0.8
Cook, only	31 (20.4%)	1970 ± 1466	21	9	42.8	2.1 ± 3.6
Do not consume	30 (19.7%)	2262 ± 1726	23	15	65.2	2.4 ± 2.1

## Supplementary Materials

The following are available online at www.mdpi.com/1660-4601/15/1/76/s1. Manganese. Figure S1: Distribution of Mn contamination in home well water on the Crow Reservation, Montana. Figure S2: Averages and standard deviations of Mn concentrations in mg/L, in home well water by ZIP code, on the Crow Reservation. Nitrate. Figure S3: Distribution of NO_3_^−^ contamination in home well water on the Crow Reservation, Montana. Figure S4: Averages and standard deviations of NO_3_^−^ concentrations in mg/L, in well water by ZIP code, Crow Reservation. Arsenic. Uranium. Geomasking.
