# Supplementary material for: Community Engaged Cumulative Risk Assessment of Exposure to Inorganic Well Water Contaminants, Crow Reservation, Montana
Source: Int J Environ Res Public Health. 2018 Jan 5;15(1):76. doi: 10.3390/ijerph15010076 (PMC5800175; doi:10.3390/ijerph15010076)
Supplement: Supplementary file 1 [file ijerph-15-00076-s001.pdf]

*Supplementary Materials*

# Community Engaged Cumulative Risk Assessment of Exposure to Inorganic Well Water Contaminants, Crow Reservation, Montana

**Margaret J. Eggers [1,2,*], John T. Doyle [2,3], Myra J. Lefthand [2], Sara L. Young [2], Anita L. Moore-Nall [4], Larry Kindness [2], Roberta Other Medicine [2,5], Timothy E. Ford [6], Eric Dietrich [1], Albert E. Parker [1,7], Joseph H. Hoover [8] and Anne K. Camper [1,2,9]**

## Manganese

Manganese exceeding the secondary standard of 0.05 mg/L is common throughout the Bighorn and Little Bighorn River Valleys (Figure S1). Averaging well water Mn concentrations by zip code shows that wells in the Hardin ZIP code on average exceed the EPA health advisory of 0.30 mg/L, and wells in the South Billings, St. Xavier and Crow Agency ZIP codes average close to or more than 50% of this health standard (Figure S2).

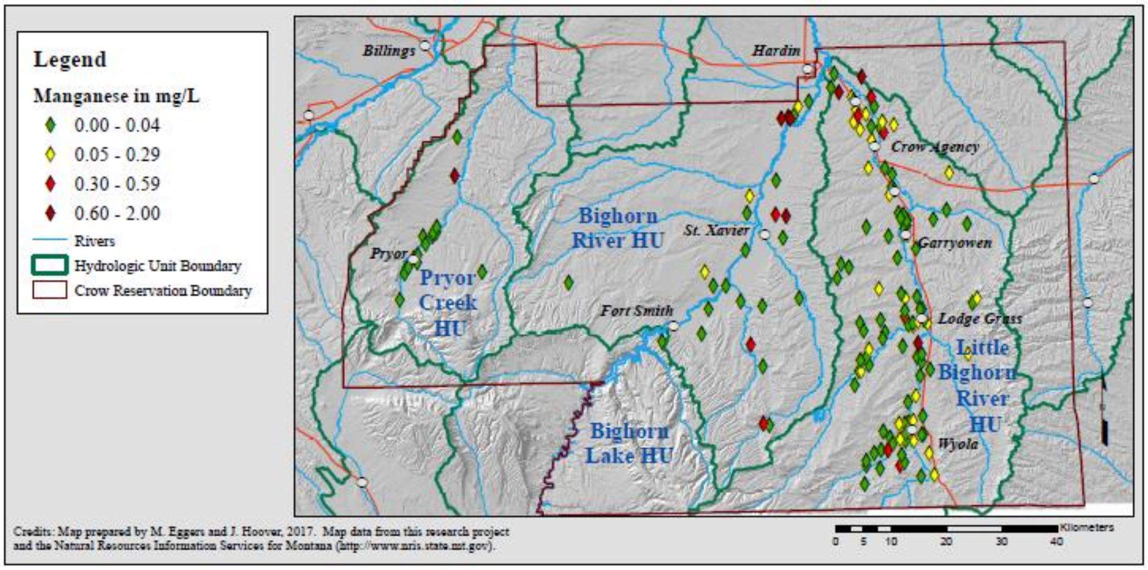


**Figure S1**. Distribution of Mn contamination in home well water on the Crow Reservation, Montana. Wells with Mn contamination at a concentration exceeding the EPA's secondary standard are shown as yellow diamonds; wells with Mn exceeding EPA's health advisory are shown as red and dark red diamonds.

| Pryor Creek | | Bighorn River Valley | | Little Bighorn River Valley | |
|---|---|---|---|---|---|
| South Billings | 0.14 ± 0.29 | Hardin | 0.35 ± 0.43 | Crow Agency | 0.14 ± 0.18 |
| Pryor | 0.005 ± 0.001 | St Xavier | 0.19 ± 0.29 | Garryowen | 0.07 ± 0.11 |
| | | Fort Smith | 0.02 ± 0.02 | Lodge Grass | 0.05 ± 0.10 |
| | | | | Wyola | 0.08 ± 0.11 |

| |
|---|
| 0 - 0.04 mg/L: Acceptable, below EPA Secondary Standard |
| 0.05 - 0.29 mg/L: Nuisance. Exceeds EPA Secondary Standard but not EPA Health Advisory. |
| ≥ 0.30 mg/L or more: Unacceptable, exceeds EPA Health Advisory. |

**Figure S2.** Averages and standard deviations of Mn concentrations in mg/L, in home well water by ZIP code, on the Crow Reservation.

**Nitrate**

The 4.3 percent of wells exceeding the EPA MCL of 10 mg/L for nitrate plus nitrite as N ("$NO_3^-$") is comparable to the 5% of wells identified by the IHS as exceeding the MCL (Table 4). Elevated $NO_3^-$ in well water was found primarily in the Bighorn River Valley, where irrigated agriculture is most extensive (Figures S3 and S4).

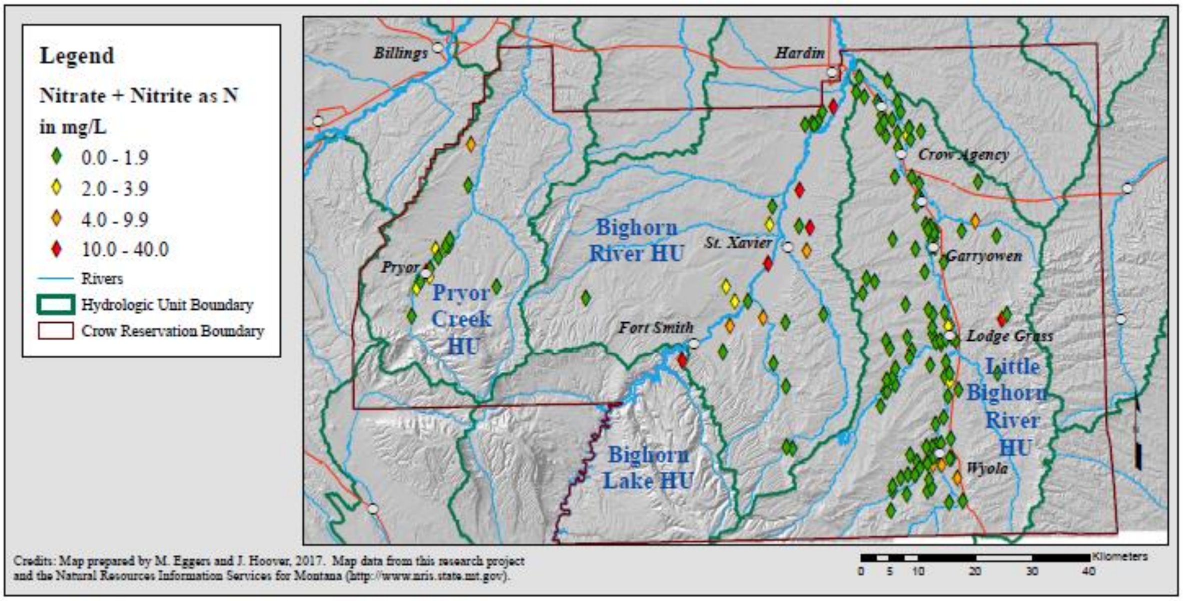


**Figure S3**. Distribution of $NO_3^-$ contamination in home well water on the Crow Reservation, Montana. Orange and red diamonds mark wells with likely anthropogenic $NO_3^-$ contamination; red diamonds indicate exceedance of the EPA MCL for nitrate + nitrite as N.

| Pryor Creek | | Bighorn River Valley | | Little Bighorn River Valley | |
|---|---|---|---|---|---|
| South Billings | 1.68 ± 2.88 | Hardin | 12.18 ± 18.89 | Crow Agency | 0.28 ± 0.52 |
| Pryor | 2.08 ± 3.55 | St Xavier | 4.8 ± 7.84 | Garryowen | 0.44 ± 0.99 |
| | | Fort Smith | 7.07 ± 8.30 | Lodge Grass | 0.20 ± 0.48 |
| | | | | Wyola | 0.66 ± 1.57 |

| |
|---|
| < 4 mg/L: Naturally occurring[a] |
| 4.0 - 9.9 mg/L: Likely contaminated by anthropogenic sources[a] |
| 10.0 mg/L or more: Exceeds EPA's MCL due to serious health risks. |

[a] http://waterquality.montana.edu/well-ed/files-images/Nitrate.pdf, accessed 12 June 2017.

**Figure S4**. Averages and standard deviations of $NO_3^-$ concentrations in mg/L, in well water by ZIP code, Crow Reservation.

**Arsenic**

Arsenic occurs primarily in Bighorn and Little Bighorn River valley wells. There are two EPA standards for As, an MCLG of 0.000 mg/L and an MCL of 0.010 mg/L. Twenty-seven percent of home wells had detectable As, i.e. they exceeded the MCLG, while only 1% exceeded the MCL (Table 4).

**Uranium**

There are two EPA standards for uranium: a maximum contaminant level goal (MCLG) of 0.000 mg/L, and a maximum contaminant level (MCL) of 0.030 mg/L. More than two thirds of wells tested positive for uranium, i.e. exceeded the MCLG (Table 4), and hence consuming this water incurs a low-level health risk. Uranium exceeding 0.030 mg/L was found in 6.2% of wells (Table 4), rendering the water from these wells unsafe for consumption. IHS data on uranium in home well water are lacking for the Crow Reservation.

Spatial analysis found that residents in the lower Bighorn River valley are most at risk: wells in the St. Xavier ZIP code average 0.018 ± 0.030 mg/L and those in the Hardin ZIP code average 0.010 ± 0.021 mg/L U. In the Crow Agency and Wyola ZIP codes of the Little Bighorn River valley, few wells exceeded the MCL, but many have elevated U concentrations. See Eggers et al. [99] for further information and a map.

**Geomasking**

While representation of point features is important for visualizing local patterns of environmental and health data, geovisualization of such information may increase the risk of participant identification because reverse geocoding methods could be used to locate study participants with derived latitude and longitude coordinates [127]. Geomasking methods have been developed in response to this risk so that data visualizations can be employed while protecting participant confidentiality [126]. Some masking methods, such as data aggregation, have low data resolution and decreased power to detect spatial patterns when the phenomenon of interest is local or crosses administrative boundaries [128]. Other classes of geomasking methods do not aggregate data and rely on random perturbation, which randomly shifts the point feature to a new location. Here a random perturbation geomasking method known as the donut method is employed, which shifts a point to a location between a minimum and maximum distance threshold and in

a random direction. This method commonly scales the distance threshold based on population density of the study area, with larger scaling in lower population areas [129].

For the visualizations created for the present study each point location was shifted a random direction and located in an area between a minimum distance ($D_{min}$) and maximum distance ($D_{max}$) from the original location:

$$D_{min} = pd^{-1} \times (a + x) \quad (S1)$$

$$D_{max} = pd^{-1} \times (b + y) \quad (S2)$$

In equations (S1) and (S2) $pd$ is the population density of the administrative district in which the point is located, $a$ and $b$ are predefined integers that ensure the point is moved a minimum distance, and $x$ and $y$ are random integers that increase the shifting distance. The minimum and maximum distance thresholds were proportional to the inverse of population density ($pd$) multiplied by a predefined minimum movement distance ($a$ or $b$) plus a random distance integer ($x$ or $y$) [126]. Population density was determined by spatially joining the US Census 2010 Census Block Group for each point; the range of $D_{min}$ and $D_{max}$ values are not reported here to maintain confidentiality. In ArcGIS (v 10.3.1; ESRI, Redland, CA, USA) the geomasking was executed using a Model Builder script. The Buffer Tool was used to generate geodesic circular buffers using $D_{min}$ and $D_{max}$ for each point. Subsequently, the minimum buffer was erased from the maximum buffer leaving a ring that defined the area of possible relocation (Figure S5). The Create Random Points Tool was used to generate one new location within each buffer ring and a spatial join was used to move the original attributes to the new location feature for visualization.

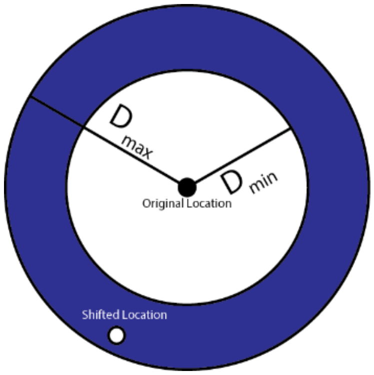


**Figure S5.** Illustration of the donut geomasking method including an example buffer ring and a hypothetical original and shifted point location.
